# Response evaluation after primary systemic therapy of Her2 positive breast cancer – an observational cross-sectional study

**DOI:** 10.3325/cmj.2015.56.128

**Published:** 2015-04

**Authors:** Tímea Tőkés, Gyöngyvér Szentmártoni, László Torgyík, Kornélia Kajáry, Zsolt Lengyel, Tamás Györke, Béla Á. Molnár, Anna-Mária Tőkés, Janina Kulka, Magdolna Dank

**Affiliations:** 1 Semmelweis University, 1st Dept. of Internal Medicine, Oncological Division, Budapest, Hungary; 2 Pozitron PET/CT Center, Budapest, Hungary; 3 Semmelweis University, Department of Nuclear Medicine, Budapest, Hungary; 4 Scanomed Ltd, Budapest, Hungary; 5 Semmelweis University, 1st Department of Surgery, Budapest, Hungary; 6 MTA-SE Tumor Progression Research Group, 2nd Department of Pathology, Budapest, Hungary; 7 Semmelweis University, 2nd Department of Pathology, Budapest, Hungary

## Abstract

**Aim:**

To evaluate (I) trastuzumab-containing primary systemic therapy (PST) in human epidermal growth factor receptor 2 (Her2) overexpressing breast carcinomas; (II) compare the patients who achieved and those who did not achieve pathological complete remission (pCR), and (III) analyze the accuracy of different clinical-imaging modalities in tumor response monitoring.

**Methods:**

188 patients who received PST between 2008 and 2014 were reviewed and 43 Her2 overexpressing breast cancer patients (28 Luminal B/Her2-positive and 15 Her2-positive) were enrolled. 26 patients received mostly taxane-based PST without trastuzumab (Group 1) and 17 patients received trastuzumab-containing PST (Group 2). We compared the concordance between pCR and complete remission (CR) defined by breast-ultrasound, CR defined by standard 18F-fluoro-deoxy-glucose positron emission tomography and computerized tomography (FDG-PET/CT) criteria (Method 1) and CR defined by a novel, breast cancer specific FDG-PET/CT criteria (Method 2). Sensitivity (sens), specificity (spec), and positive (PPV) and negative predictive values (NPV) were calculated.

**Results:**

10 patients (38.5%) in Group 1 and 8 (47%) in Group 2 achieved pCR. pCR was significantly more frequent in Her2-positive than in Luminal B/Her2-positive tumors in both Group 1 (*P* = 0.043) and Group 2 (*P* = 0.029). PET/CT evaluated by the breast cancer specific criteria (PET/CT Method 2) differentiated pCR from non-pCR more accurately in both groups (Group 1: sens = 77.8%, spec = 100%, PPV = 100%, NPV = 71.4%; Group 2: sens = 87.5%, spec = 62.5%, PPV = 70%, NPV = 83.3%) than standard PET/CT criteria (Method 1) (Group 1: sens = 22.2% spec = 100% PPV = 100% NPV = 41.7%; in Group 2: sens = 37.5%, spec = 87.5%, PPV = 75% NPV = 58.3%) or breast ultrasound (Group 1, sens = 83.3% spec = 25% PPV = 62.5% NPV = 50%; Group 2, sens = 100% spec = 12.5% PPV = 41.6% NPV = 100%).

**Conclusion:**

The benefit of targeted treatment with trastuzumab-containing PST in Her2 overexpressing breast cancer was defined in terms of pCR rate. Luminal B/Her2-positive subtype needs further subdivision to identify patients who would benefit from PST. Combined evaluation of tumor response by our novel, breast cancer specific FDG-PET/CT criteria accurately differentiated pCR from non-pCR patients.

The initial main goal of primary systemic therapy (PST, also known as neoadjuvant therapy) was to allow surgical intervention in locally advanced breast cancer and inflammatory breast cancer by downstaging (1-5). However, it led to favorable clinical response rates (reaching 65%) and pathological complete remission (pCR) rates (between 4%-29%) (6), and patients achieving pCR showed significantly longer disease-free and overall survival than non-responders (7-9). Based on these results, achievement of pCR became the primary endpoint of PST. Moreover, introduction of trastuzumab, the first targeted agent against human epidermal growth factor receptor 2 (Her2), in the PST setting improved the pCR rate and resulted in longer disease-free survival in Her2 overexpressing breast cancers (10,11).

In Hungary, trastuzumab-containing PST for Her2 overexpressing breast cancers has been routinely available since 2013. The aims of our study are:

(I) to evaluate the benefit of personalized, trastuzumab-containing PST regimens in daily routine practice compared to treatments without this agent.

(II) to compare patients who after the therapy (with or without trastuzumab) achieved pCR and those who did not (non-pCR ~ any form of residual disease after PST).

(III) to compare the accuracy of clinical-imaging tests to assess tumor response after PST by using breast ultrasound and fluoro-deoxy-glucose positron emission tomography and computerized tomography (FDG-PET/CT). 

Our third hypothesis is based on the fact that initiating a personalized and targeted treatment approach calls for accurate monitoring of treatment response. By using reliable predictive factors for tumor response, oncologists will be able to adapt and modify therapeutic regimens during PST to achieve pCR more frequently and improve clinical outcomes (1-4). At first, upon administration of PST for breast cancer, local extension of the tumor and therapeutic response were measured routinely with conventional imaging techniques like breast ultrasound (2). Over the past decade FDG-PET/CT imaging proved suitable for breast cancer staging as well as for response evaluation during PST (12-14). However, only a limited number of studies are available on the application of FDG-PET/CT in Her2 overexpressing breast carcinomas and its accuracy is questionable when targeted, biological therapies are administered (15-21).

To improve the accuracy of end-therapy imaging, we evaluated the FDG-PET/CT scans not just by standard metabolism-based criteria but also by a novel, combined imaging analysis method. In the case of breast malignancy there are no specific response criteria to assess the tumor response to PST besides the standard generalized PET Response Criteria in Solid Tumors (PERCIST) criteria (22). In PERCIST, morphology is considered as relevant in case of disease progression, but not if complete remission (CR) is evaluated. We wanted to develop an evaluation method that combined the metabolism and morphology-based tumor response criteria to define CR, but in a more simplified and less time-consuming manner. We compared the applicability of the standard and novel methods in Her2 overexpressing tumors treated with standard chemotherapy as well as with targeted, trastuzumab-containing PST.

## Patients and methods

### Patients

Patients diagnosed with primary breast cancer and treated with PST at the Oncological Division of the 1st Department of Internal Medicine of the Semmelweis University between 2008 and 2014 were retrospectively identified. The diagnosis of breast cancer was confirmed by core biopsy. After completion of PST, all patients underwent surgery. Ethical approval for the study was given by Semmelweis University Institutional Review Board and written informed consent was waived (SE. TUKEB 120/2013).

### Histopathological analysis and pathological response evaluation

**Figure 1:**
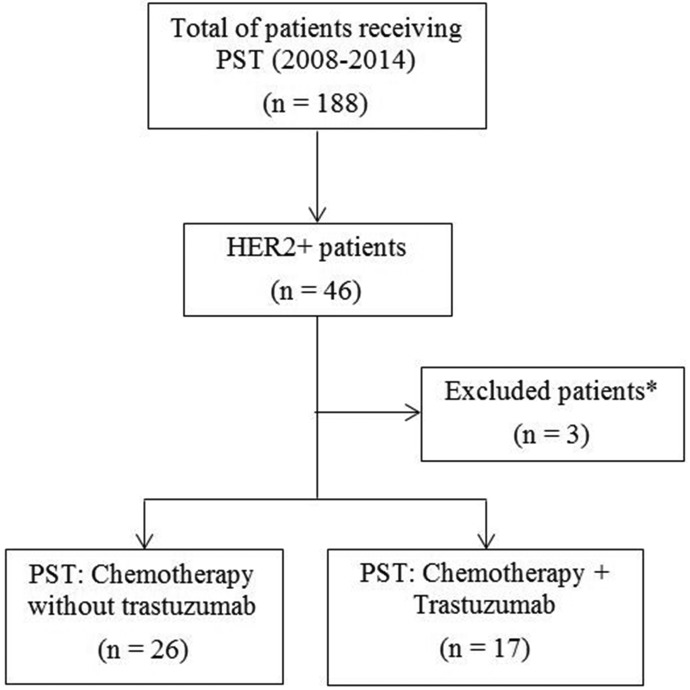
Study flow-chart showing the number of patients with Her2 overexpressing tumors (Her2+) among those receiving primary systemic therapy (PST). *Reason for exclusion: PST was not completed.

Histological analysis of tumor tissues was performed routinely on core biopsy specimens before therapy and on surgical samples after the PST. On core biopsy samples, detailed histological characterization was performed (histological type, nuclear grade, tubule formation, mitotic index, inflammatory cell infiltrate, presence or absence of in situ carcinoma component and lymphovascular invasion). On surgical samples, pCR was diagnosed only if no viable invasive tumor cells were identified after the whole tumor bed was embedded and thoroughly investigated. If residual tumor was present, the detailed histological characterization was repeated and tumor size and nodal stage were assessed. Immunohistochemistry (IHC) was performed on paraffin-embedded tissue samples to evaluate hormone receptor (HR) (estrogen or progesterone), Her2 expression, Ki-67 labeling index (Ki-67 LI), and p53 tumor suppressor protein. HR positivity was confirmed if the Allred score was above or equal to 3 (23). Only Her2 positive patients were included in the current analysis (Figure 1). Her2 overexpression was defined as IHC 3+. For IHC 2+ samples, fluorescent in situ hybridization (FISH) was performed to confirm gene amplification. Her2 1+ or 0 tumors were considered as Her2 negative and were excluded from the analysis. Her2 status was defined according to the ASCO/CAP Guideline valid at the time of diagnosis, ie, Her2-positive patients treated between 2008 and November 2013 were identified according to the 2007 ASCO/CAP Guideline (24), and from then on according to the Guideline published in October 2013 (25).

Biological subtypes of tumors were defined according to the recommendations of the 13th St. Gallen International Breast Cancer Conference (26) as follows: Luminal B-like Her2 positive tumors (Luminal B/Her2-positive) were defined by immunohistochemistry as both Her2 and HR positive and Her2-positive subtypes were defined as Her2 positive and HR negative. 

Patients who achieved pCR were identified according to national consensus recommendations (23) based on the Pinder response classification (27). Briefly, the definition of pCR was the following: no residual tumor tissue in the surgical samples, but presence of ductal carcinoma in situ was allowed.

### Clinical response evaluation

For local staging of primary tumors, physical examination, breast ultrasound, and x-ray mammography were performed. To evaluate the presence of distant metastases, FDG-PET/CT scans were performed. If PET/CT was not available or applicable, chest, abdominal, and pelvic CT with bone scintigraphy was chosen for staging. Clinical TNM stage was determined according to the 7th edition of American Joint Committee on Cancer classification (5). After PST, the tumor response of primary breast lesions was evaluated by PET/CT (if PET/CT was used for staging) or by ultrasound alone.

### Ultrasound evaluation

Breast ultrasound (Esaote MyLab ^TM^ 25, Esaote North America, Indianapolis, IN, USA; Philips HD 15^TM^, Philips Healthcare, Andover, MA, USA) was performed routinely before and after PST by two experienced breast-radiologists. The same radiologist performed all the tests for the same patient to avoid inter-observer variability and consequential bias; otherwise ultrasound results were excluded from the analysis. Target breast lesions (longest diameter) were measured in every session. CR was defined as no sign of residual tumor tissue by ultrasound after the last cycle of PST.

### PET/CT response

PET/CT scans were performed with dedicated whole-body PET/CT scanners (Siemens Biograph^TM^ TruePoint^TM^ HD, Siemens Healthcare, Malvern, PA, USA; GE Discovery^TM^ ST 8, GE Healthcare, Waukesha, WI, USA) following standard protocols and guidelines to ensure the highest reproducibility and comparability (22,28-30). The author who analyzed PET/CT images was blinded to the patient’s clinical records (results of conventional imaging). The regions of interests were located manually over the primary tumor (31,32). Two types of response evaluations were performed:

(1) Method 1***:*** On FDG-PET/CT scans, maximum of the Standardized Uptake Value (SUVmax) was measured. Based on SUVmax (weights of patients were relatively stable during the study period), PERCIST criteria were applied to define CR. The PERCIST-based definition of complete remission is the following: complete resolution of 18F-FDG uptake within measurable target lesion with the disappearance of all other lesions to background blood-pool levels and without new 18F-FDG-avid lesions in pattern typical of cancer. In the PERCIST-defined CR criteria, Response Evaluation Criteria in Solid Tumors (RECIST)-based evaluation of tumor morphology is only considered in the case of progression (22). In our study, cases with partial remission, stable disease, or tumor progression were simply categorized as tumors with non-complete remission (non-CR).

(2) Method 2: Additional, novel evaluation of PET/CT examinations was performed. Morphological tumor remission was defined according to RECIST (Response Evaluation Criteria in Solid Tumors, v1.1) for CT (33). All primary breast lesions were initially measurable on CT by RECIST and were suitable for response evaluation by this method. CR was defined as both metabolic (CR defined by PERCIST) and morphological (CR defined by RECIST) CR. If either criterion (PERCIST or RECIST) showed residual disease (ie, partial remission, stable disease, or progression), the response could not be classified as CR.

### Correlation with pathological response

The breast ultrasound-based and the PET/CT-based CR rates – defined by Method 1 and Method 2 – were compared with the pCR rates after the PST to assess the accuracy of the applied imaging modalities and response evaluation methods.

### Statistical analysis

Data are expressed as mean ± standard deviation (SD). Mann-Whitney tests were used to compare response evaluation markers and tumor characteristics of pCR and non-pCR groups. For binomial data, contingency tables were constructed and Fisher exact tests were applied due to the relatively low number of cases in each category. All applied statistical tests were two-sided, and *P*-values <0.05 were considered significant. To measure the accuracy of the applied clinical response criteria, we calculated sensitivity, specificity, and positive and negative predictive values of the diagnostic tests. For data collection and processing we used Microsoft Excel 2010 (Microsoft Corp. Redmond, WA, USA), Statistica 64 11 (StatSoft Inc., Tulsa, OK, USA) and MedCalc 13.2.2 (*http://www.medcalc.org/*) software.

## Results

### Patient characteristics and treatment schedules

Among 188 breast cancer patients who underwent PST, there were 46 Her2 overexpressing tumors and 43 patients were enrolled in the study (Figure 1). Among the 43 patients,(age 51.47 ± 11.07 years), 15 had Her2-positive subtype and 28 Luminal B/Her2-positive breast cancer. All patients were treated with PST, most commonly in 3 week schedules, for 6-8 cycles. 26 patients received mostly taxane-based PST and adjuvant trastuzumab treatment (Group 1) and 17 patients received a trastuzumab-containing, taxane-based PST protocol (Group 2) (Table 1). After PST, every patient gave consent to surgery: 25 patients (58.1%) underwent mastectomy and 18 (41.9%) had breast-conserving surgery (sector resection of the breast or quadrantectomy), with 40 axillary block dissections (93%) and 3 sentinel lymph node biopsies (7%). Re-excision was not necessary.

**Table 1:** Patient and tumor characteristics (n = 43)*

Characteristics	No.	Percent
**Age**		
<30 years	2	4.6
30–39 years	3	7
40–49 years	12	28
50–59 years	17	39.5
≥60 years	9	20.9
**Menopausal status**		
premenopausal	18	41.9
perimenopausal	23	53.5
postmenopausal	2	4.6
**Clinical T stage**		
T1c	4	9.3
T2	28	65.1
T3	5	11.6
T4	6	14
**Clinical N stage**		
N0	19	44.2
N1	18	41.9
N2	2	4.6
N3	4	9.3
**Histology**		
invasive ductal carcinoma	37	86.1
invasive lobular carcinoma	2	4.6
other	4	9.3
**Grade^†^**		
2	13	30.2
3	28	65.1
**Biological subtype**		
Her2-positive	15	34.9
Luminal B/Her2-positive	28	65.1
**Estrogen receptor status**		
positive	27	62.8
negative	16	37.2
**Progesterone receptor status**		
positive	25	58.1
negative	18	41.9
**Her2 status**		
positive	43	100
**Ki-67 LI**^‡§^		
high	35	81.4
low	6	13.9
**p53 status^║^**		
positive	24	55.8
**Treatment regimen – Group 1 (n = 26)**		
docetaxel + carboplatin	9	34.6
docetaxel + epiadriamycin	6	23.1
doxorubicin + paclitaxel	3	11.5
5-fluorouracil + epirubicin + cyclophosphamide	2	7.7
docetaxel + doxorubicin + cyclophosphamid	6	23.1
**Treatment regimen – Group 2 (n = 17)**		
trastuzumab + Docetaxel 4x → FEC 4x	9	53
trastuzumab + Docetaxel + carboplatin	4	23.5
trastuzumab + Docetaxel	4	23.5

*FEC – 5-fluorouracil + epirubicin + cyclophosphamide; Her2 – human epidermal growth factor receptor 2; LI – labeling index.

^†^Grade 1: 0, Unknown: 2 patients.

^‡^Unknown: 2 patients.

^§^Cut-off: 14%.

^║^Unknown: 3 patients.

### Tumor remission and response evaluation

All 43 primary tumors were morphologically measurable at the time of diagnosis (both with ultrasound and CT by RECIST 1.1 criteria, if applicable) (33) and metabolically active on the FDG-PET/CTs. FDG-PET/CT was performed to measure tumor remission in 14 patients from Group 1 and 16 from Group 2. Results of ultrasound measurements regarding local extension and its changes were available in both groups (20 and 13 patients in Group 1 and 2, respectively).

Favorable response to PST was observed in both groups. 10 patients (38.5%) from Group 1 and 8 (47%) from Group 2 showed pCR. Tumors belonging to the Her2-positive subtypes showed pCR significantly more frequently than Luminal B/Her2-positive tumors (*P* = 0.043 and *P* = 0.029, respectively). Out of the 15 Her2-positive tumors, 11 showed pCR: 7 in Group 1 and 4 in Group 2. Of the 28 Luminal B/Her2-positive tumors, only 7 showed pCR: 3 in Group 1 and 4 in Group 2 (Table 2).

**Table 2:** Subgroup analysis of patients who achieved pathological complete remission (pCR) and those who did not (non-pCR) in Group 1 and Group 2*

	Group 1			Group 2		
	N	Mean ± SD	*P*	N	Mean ± SD	*P*
**Histological characteristics**						
Ki-67 LI (%)						
pCR	9	52.7 ± 25.1	0.16	8	32.5 ± 13.7	0.45
non-pCR	16	37.4 ± 23.6		8	29.4 ± 18.8	
Grade^†^						
pCR^‡^	grade 2 = 4 grade 3 = 5		0.67	grade 2 = 0 grade 3 = 9		0.07
non-pCR^§^	grade 2 = 5 grade 3 = 11			grade 2 = 4 grade 3 = 4		
Subtype^†^						
pCR	Luminal B/Her2-pos. = 3 Her2-positive = 7		**0.04**	Luminal B/Her2 pos. = 4 Her2-positive = 4		**0.03**
non-pCR	Luminal B/Her2-pos. = 12 Her2-positive = 4			Luminal B/Her2 pos. = 9 Her2-positive = 0		
**Response markers**						
SUVmax1						
pCR	5	27.1 ± 17.8	**0.02**	8	13.1 ± 6.4	0.37
non-pCR	9	12.1 ± 4.9		8	9.8 ± 4.2	
SUVmax2						
pCR	5	1.4 ± 0.7	0.59	8	1.5 ± 0.5	0.10
non-pCR	9	1.9 ± 1.4		8	3.4 ± 3.2	
SUVmax changes						
pCR	5	92.7 ± 5.5	0.14	8	84.5 ± 11.8	0.05
non-pCR	9	80 ± 16.2		8	64 ± 28.8	
Size 1 (mm)						
pCR	10	34.3 ± 14.6	0.38	8	29.6 ± 7.1	0.74
non-pCR	16	28.7 ± 13.3		9	36.9 ± 18.1	
Size 2 (mm)						
pCR	8	9.7 ± 8.7	0.17	8	10.7 ± 6.9	0.12
non-pCR	12	15.6 ± 9.9		5	22.6 ± 16.2	
Size changes						
pCR	8	74.1 ± 20.31	0.07	8	64.8 ± 26.6	0.12
non-pCR	12	46.8 ± 35.1		5	38.9 ± 21.2	

*SUVmax 1 and 2 – maximum of the standardized uptake value before (SUVmax 1) and after (SUVmax 2) the primary systemic therapy. Ki-67 LI – Ki-67 labeling index.

†Fisher exact test results. Otherwise: Mann-Whitney test. Significant results in bold.

^‡^Unknown: in 1 patient from Group 1.

^§^Unknown: in 1 patient from Group 2.

In Group 1 the initial FDG uptake was significantly higher in pCR than in non-pCR patients. Other parameters of tumor metabolism, the results of morphological measurements (tumor size before or after PST or changes in size), and initial tumor proliferation activity (core-biopsy Ki-67 LI) were not significantly different between pCR and non-pCR patients (Table 2).

The accuracy of PET/CT and breast ultrasound for clinical discrimination of pCR-/non-pCR groups was also measured (Table 3). The ultrasound results in both groups followed the same pattern: the number of false positive cases was considerably high (6 cases in Group 1 and 7 cases in Group 2), while PPV (62.5% in Group 1 and 41.7% in Group 2) and specificity (25% in Group 1 and 12.5% in Group 2) were low. However, false negativity was also low (2 cases in Group 1 and 0 in Group 2).

**Table 3:** Diagnostic test evaluation for breast ultrasound and PET/CT evaluated by Method 1 and Method 2*

	Ultrasound		PET/CT Method 1		PET/CT Method 2	
	Group 1 (n = 20)	Group 2 (n = 16)	Group 2 (n = 14)	Group2 (n = 16)	Group 1 (n = 14)	Group 2 (n = 16)
Sensitivity (%) (95% CI)	83.3 (51.6-97.4)	100 (47.9-100)	22.2 (3.5-59.9)	37.5 (9-75.3)	77.8 (40.1-96.5)	87.5 (47.4-97.9)
Specificity (%) (95% CI)	25.0 (3.4-64.9)	12.5 (2.1-52.6)	100 (47.9-100)	87.5 (47.4-97.9)	100 (47.9-100)	62.5 (24.7-91)
Positive predictive value (%) (95% CI)	62.5 (35.5-84.7)	41.7 (15.3-72.2)	100 (19.3-100)	75 (20.3-95.9)	100 (58.9-100)	70.0 (34.8-92.9)
Negative predictive value (%) (95% CI)	50.0 (8.3-91.7)	100 (16.5-100)	41.7 (15.3-72.2)	58.3 (27.7-84.7)	71.4 (29.3-95.5)	83.3 (36.1-97.2)
False positive (No.)	6	7	0	1	0	3
False negative (No.)	2	0	7	5	2	1

*PET/CT – Positron Emission Tomography and Computerized Tomography; CI – confidence interval.

Evaluation of tumor response by PET/CT with Method 1 showed a higher number of false negative cases compared to ultrasound (7 cases in Group 1 and 5 cases in Group 2). However, the number of false positive cases was very small (0 cases in Group 1 and 1 case in Group 2); PET successfully detected the residual disease.

Evaluation of tumor response by PET/CT with Method 2 had high sensitivity (77.8% in Group 1 and 87.5% in Group 2) and high specificity (100% in Group 1 and 62.5% in Group 2). The number of false negative cases was low (0 in Group 1 and 3 in Group 2) and NPV was considerably higher compared to Method 1 (Group 1: 71.4% vs 41.7%; Group 2: 83.3% vs 58.3%).

## Discussion

In Her2 overexpressing breast cancer, adding trastuzumab to chemotherapy regimens during PST improved clinical outcomes and resulted in higher rates of pCR (11,34-38). In agreement with these reports, we showed that the pCR rate after PST with concomitant trastuzumab was higher (47%, Group 2) than in the patient group that did not receive additional trastuzumab therapy during the PST (Group 1, with a pCR rate of 38.5%). Therefore, we confirmed the clinical benefit of trastuzumab-containing PST in the daily routine; moreover, trastuzumab performed even better in the daily practice than it did in clinical trial conditions – the pCR rate in our study was 47% in the trastuzumab arm, which is better than the 43% achieved in the NOAH trial (11) or the 31.7% pCR rate in the GeparQuattro study (37). We also confirmed that in the case of Her2 overexpressing breast cancer, trastuzumab should be part of the PST, and not just administered adjuvantly.

While analyzing the main characteristics of the breast tumors we compared the patients who achieved pCR and those who did not. In contrast to an earlier report (39), pCR and non pCR patient groups in our study did not show any difference in the initial Ki-67 LI. Moreover, we did not detect significant differences between grade 2 and 3 tumors, although all patients achieving pCR in Group 2 had grade 3 carcinomas.

In our study, Her2-positive tumors achieved pCR more frequently than Luminal B/Her2-positive subtypes. This is in agreement with an earlier report that suggested different clinical behavior of these tumor subtypes (40). Luminal B/Her2-positive group is a rarely investigated but important subgroup of Her2 overexpressing tumors, for which targeted therapy could be applied during the PST. While in the Her2-positive subtypes, pCR is predictive for favorable clinical outcome, in the Luminal B/Her2-positive subgroup it might not be a surrogate endpoint and might not be associated with improved disease-free survival (41). Until this question remains obscure, in case of Luminal B/Her2-positive, primarily resectable disease, clinical oncologists should consider choosing surgical treatment instead of PST as first therapeutic approach. Consequently, if Luminal B/Her2-positive subgroup less frequently achieves pCR, the indication for PST for these patients should only be downstaging of the disease (to increase the number of patients eligible for breast-conserving surgery) (3,42-44). This clinical approach should be considered until we are able to subdivide the Luminal B/Her2-positive subgroup to detect those patients who would surely achieve pCR, thus bearing the survival benefit of PST. Further randomized clinical trials with a larger cohort are needed, and the subdivision and differentiation should be based on a reliable imaging modality or biomarkers However, if we decide to apply different therapeutic protocols to different tumor subtypes, we risk the bias of the initial core biopsy sampling: biopsy results could be misguided by tumor heterogeneity and sampling methodology. A suitable tool to measure this heterogeneity and guide the biopsy sampling could be PET/CT (45).

Our study showed that the initial FDG-uptake of tumors (SUVmax1 measured before the PST) was significantly higher in pCR group than in non- pCR group, but only in Group 1. However, change in SUVmax showed no significant difference between pCR and non-pCR patients, and only a slight difference was detected in Group 2. These findings underline the previous results (19,21), which suggested that the change in SUVmax did not correlate with pCR in Her2 overexpressing tumors. This is contrary to the findings in triple negative breast cancers, when changes in the FDG-uptake correlated well with the achieved pathological remission rate (46).

A limited number of studies are available on the application of FDG-PET/CT in Her2 overexpressing breast carcinomas (15-21). The rationale behind our study was the emerging role of hybrid imaging technologies for response evaluation during PST, but there is a lack of experience in this particular patient group, especially when treated with targeted anti-Her2 therapy. The suitability of FDG-PET/CT has already been proven in breast cancer (12-14), but its efficacy depends on several tumor-properties, for instance histological tumor type (invasive ductal carcinomas are better candidates for PET/CT examinations than invasive lobular cancers) or proliferation rate (high Ki-67 LI is favorable in this respect) (47-49). FDG-uptake is also influenced by biological subtypes; Her2-positive carcinomas (and triple negative, especially basal like tumors) (24) show higher FDG-uptake than hormone receptor positive ones (50,51).

The accuracy of response evaluation with FDG-PET/CT in Her2 overexpressing breast carcinomas is contested. Small animal PET had a high positive predictive value for evaluation of tumor response to trastuzumab therapy in preclinical settings (15). However, in clinical research PET/CT was less accurate, and a possible inflammatory response induced by trastuzumab was assumed, which could have resulted in false positivity during PET imaging (16). Trastuzumab also seemed to have an effect on cellular glucose metabolism with a possible reduction of glucose uptake and consumption and FDG-incorporation (17). New tracers, especially radiopharmaceutically labeled (pl. ^89^Zr) trastuzumab or its fragments, are good candidates for PET/CT imaging during anti-Her2 therapy (18) and could possibly resolve the above mentioned bias of FDG-based PET/CT imaging. Nevertheless, PET/CT imaging proved to be highly predictive for pCR by Groheux et al (19), Hatt et al (20), and Humbert et al (21), even after one or two administered cycles of PST. Apart from these favorable results and the expanding application of FDG-PET/CT in daily oncological practice, our results underline the importance of a novel, combined metabolism and morphology-based response evaluation system in Her2 overexpressing breast carcinomas for CR.

In our study, the conventionally and routinely applied breast ultrasound poorly identified residual tumors and appeared to be inferior for response evaluation after the PST than PET/CT imaging – evaluated by both methods. Neither the breast ultrasound-based nor the PERCIST-based definitions of CR (PET/CT with Method 1) were accurate enough to predict pCR. The novel, combined definition (PET/CT with Method 2) – based on PERCIST and RECIST criteria – accurately separated pCR and non-pCR patients, in both treatment groups. These combined criteria more accurately confirmed residual disease and more specifically identified pCR. These results support the hypothesis based on our earlier results: RECIST criteria should be included in the therapeutic response evaluation criteria of breast cancers after PST (52).

The main limitation of our study was the relatively low number of patients, due to the limited availability of neoadjuvant trastuzumab treatment in Hungary at the time of the study. The number of patients prevented further differentiation of cases to analyze the deeper molecular mechanism involved in PET/CT imaging to explore differences in the FDG-consumption between Her2-positive and Luminal B/Her2-positive subtypes. However, Groheux and Humbert did not find significant differences between the FDG-consumption in these two patient groups (19,21), which is why we did not consider it necessary to address this issue in the present study. In addition, CT response evaluation plays an important role in our study, although it has limited accuracy in breast tissue. However, in our study all tumors evaluated by RECIST criteria were confirmed to be morphologically measurable at the time of the initial PET/CT, justifying the application of this method.

In summary, in Hungary PST treatments with trastuzumab are now part of the daily routine. The benefit of these regimens is visible in Her2 overexpressing tumors compared with previous regimens without trastuzumab that used pCR as primary endpoint. However, our findings suggest a possible association between biological subtypes and clinical outcome of PST. In the Luminal B/Her2-positive subgroup pCR was less frequent than in the Her2-positive subtype. This subgroup might need further subdivision using clinical biomarkers to identify those Luminal B/Her2 positive patients who would achieve pCR. In this patient group, PST should only be considered for downstaging the disease to reach operability or to support a breast-conserving surgical approach.

In conclusion, response evaluation after PST in Her2 overexpressing tumors with a metabolism based imaging technique (PET/CT) outperformed the applied conventional imaging methods (breast ultrasound). Moreover, our novel PET/CT response criteria, which comprise the PERCIST and RECIST criteria for defining CR, accurately separated pCR and non-pCR patients and were easy to apply in the daily practice.
